# 3D Shape-Weighted Level Set Method for Breast MRI 3D Tumor Segmentation

**DOI:** 10.1155/2018/7097498

**Published:** 2018-06-13

**Authors:** Chuin-Mu Wang, Chieh-Ling Huang, Sheng-Chih Yang

**Affiliations:** ^1^Department of Computer Science and Information Engineering, National Chin Yi University of Technology, Taichung 41170, Taiwan; ^2^Department of Interaction Design, Chang Jung Christian University, Tainan 71101, Taiwan

## Abstract

Three-dimensional (3D) medical image segmentation is used to segment the target (a lesion or an organ) in 3D medical images. Through this process, 3D target information is obtained; hence, this technology is an important auxiliary tool for medical diagnosis. Although some methods have proved to be successful for two-dimensional (2D) image segmentation, their direct use in the 3D case has been unsatisfactory. To obtain more precise tumor segmentation results from 3D MR images, in this paper, we propose a method known as the 3D shape-weighted level set method (3D-SLSM). The proposed method first converts the LSM, which is superior with respect to 2D image segmentation, into a 3D algorithm that is suitable for overall calculations in 3D image models, and which improves the efficiency and accuracy of calculations. A 3D shape-weighted value is then added for each 3D-SLSM iterative process according to the changes in volume. Besides increasing the convergence rate and eliminating background noise, this shape-weighted value also brings the segmented contour closer to the actual tumor margins. To perform a quantitative analysis of 3D-SLSM and to examine its feasibility in clinical applications, we have divided our experiments into computer-simulated sequence images and actual breast MRI cases. Subsequently, we simultaneously compared various existing 3D segmentation methods. The experimental results demonstrated that 3D-SLSM exhibited precise segmentation results for both types of experimental images. In addition, 3D-SLSM showed better results for quantitative data compared with existing 3D segmentation methods.

## 1. Introduction

In the process of breast cancer screening or medical treatment, the size and shape of a tumor is often an important basis for the diagnosis or treatment strategy. Segmenting the tumor from a medical image can improve the diagnostic accuracy of doctors, and become a guide for the surgery. In the past, many two-dimensional (2D) image segmentation techniques have been developed, such as the active contour model (ACM) [1], region growing, zero crossing [2], thresholding [3], region-based segmentation [4], watershed [5], fuzzy c-means (FCM), texture features, and the level set method (LSM) [6, 7]. However, breast MRI has a relatively low resolution, and tumor boundaries are often indistinct as tumors infiltrate surrounding healthy tissue. Consequently, breast MRI tumor segmentation has always been a challenging task. According to the literature on image segmentation, some segmentation methods are based on the brightness; however, these methods are easily affected by noise. Some methods are gradient-based and may result in errors if the boundaries are not clear. In addition, some methods are based on local features; however, they are often dependent on the suitability of the features extracted as well as the image consistency. If these methods are used in highly variable medical images, their segmentation accuracy is also relatively unstable. However, some segmentation methods demonstrate superior performance, such as LSM and multispectral detection technology [7] that do give good results for tumor segmentation in 2D medical images. It is noteworthy that in order to meet the needs of multispectral detection technology, different parameters (such as T1, T2, and PD) must be used to produce multispectral MRIs.

Using three-dimensional (3D) models would undoubtedly determine tumor size and shape more accurately than using 2D imaging. However, most of the current medical imaging instruments present only 2D images. Although there are some relatively expensive instruments that can stack the original 2D images directly into a 3D model, the original 2D images usually contain mixed complex background tissues, often making the object difficult to recognize in 3D. Therefore, the segmentation of 3D medical images has become a computer-aided diagnostic technology in dire need of development [8]. Due to the technologies of 2D image segmentation and contour detection are now relatively mature, some 3D image segmentation methods use 2D segmentation as the foundation to carry out segmentation on 2D sections before stacking these 2D segmentation results into a 3D segmentation model [9–11]. As these 3D segmentation methods lack association between upper and lower sections (*Z*-axis), the accuracy of these methods in 3D segmentation applications is not as high as in 2D segmentation. In addition, as these methods calculate every 2D section one-by-one, and not the total 3D calculations, the computational burden is significantly increased. At present, there are some existing 3D image segmentation techniques. For example, Rebouças et al. [12] developed ACM into 3D-ACM and compared it with 3D region growing. Although the experimental results showed that 3D-ACM had the better performance, it was closely related to the settings of the initial contour and was difficult to use as a clinical diagnostic aid. In addition, Gangsei and Kongsro [11] extended Dijkstra's algorithm to a 3D algorithm, and conducted 3D segmentation for bone CT images. Despite the fact that satisfactory segmentation results were obtained, this method is not suitable for use in breast MRI with its various mixed tissues and low contrasts. This is because there is a high contrast between the target and background tissues in bone and vascular imaging, a fact that is exploited by other 3D image segmentation algorithms [13, 14] for bones and blood vessels. Some methods carry out segmentation at three orthogonal 2D planes before combining these segmentation results into a 3D segmentation model [14, 15]. Although these methods significantly decrease the computational burden, they are still based on 2D segmentation and are not strictly true 3D segmentation methods.

Based on the aforementioned analyses, this paper proposes a new 3D tumor segmentation method, namely, the 3D shape-weighted level set method (3D-SLSM). In comparison with past methods as well as our previous research results in [9], 3D-SLSM has three major advantages. Firstly, it is evolved from 2D-LSM, whose tissue segmentation has been confirmed to give good results for 2D breast MRI [7]. Secondly, 3D-SLSM operates directly on the entire 3D model, which not only reduces the computation time but also ensures the association and interaction between each pixel and its neighboring points on all three axes (*X*, *Y*, and *Z*). Thirdly, 3D-SLSM adds a characteristic shape-weighted model in each update, so that the contour converges rapidly towards the surface of the target object, effectively eliminating the mixed surrounding background noise. In order to verify experimentally the feasibility of the proposed method, experimental data are assigned to two groups: computer-simulated images and breast MRI cases with actual tumors. Computer-simulated images help us to observe based on quantitative analysis while breast MRIs are used to confirm efficiency in clinical applications. In the course of the experiment, the segmentation of experimental images using various algorithms will be carried out. However, as the 3D segmentation results of the various algorithms are 3D point matrices that cannot be directly viewed, rendering techniques must be first employed to convert these 3D point matrices into visual 3D image models to facilitate observation and actual applications. A quantitative evaluation is then conducted using a standard model (delineated by physicians for actual MRI cases) as a basis. Besides evaluating the performance of 3D-SLSM, the accuracy and error rates of 3D-SLSM are also compared to existing algorithms, such as traditional ACM and 2D/3D-LSM, in order to validate the contributions of 3D-SLSM.

This paper is divided into five sections, which are as follows: Section 2 introduces the new 3D-SLSM method proposed in this paper.  Section 3 describes the reconstruction and system evaluation methods for experimental data.  Section 4 presents experimental results and discussion. Finally, conclusions are presented in Section 5.

## 2. Methods

### 2.1. Three-Dimensional Level Set Method (3D-LSM)

**Figure 1:**
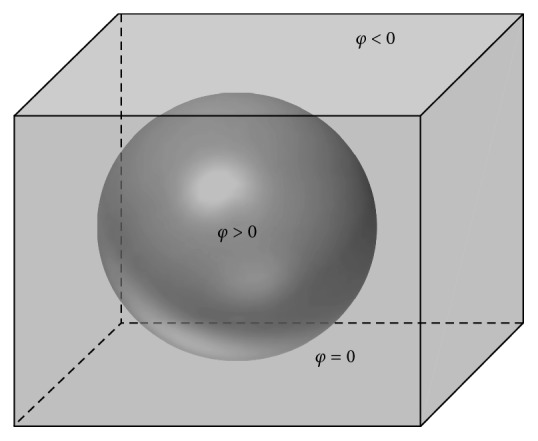
Initial contour surface of the 3D level set method.

The traditional LSM was first proposed in 1988 by Osher and Sethian [16], and it is still used widely in many disciplines today. LSM has already been confirmed to have superior performance in the segmentation of 2D medical images. Therefore, while developing 3D-SLSM, we selected LSM as a foundation, and first converted traditional 2D-LSM into 3D-LSM, which can be used for overall calculations in 3D models. In addition to increasing the computational efficiency, this conversion also significantly increases the accuracy of 3D segmentation as the computation process considers the association of pixels on upper and lower sections (*Z*-axis). Conventional 2D-LSM calculations require the construction of an initial 2D target region, and *φ* is used to express the height level of every image pixel. After the boundaries of the initial target region were taken as horizontal lines (*φ* = 0), a height distribution map is then constructed according to the characteristics of the various positions in the images. The proposed 3D-LSM first upgrades the 2D initial target region into a 3D initial target object before taking the contour surfaces of the initial target object as horizontal planes. *φ* = 0 indicates that the point is on its contour surface, *φ* > 0 indicates that the point is located inside the target, and *φ* < 0 indicates that the point is located outside the target, as shown in Figure 1.

In order to increase the association of the *Z*-axis for conversion into a 3D algorithm, a new formula was redefined for 3D-LSM, as shown in the following equation:

(1) $$\begin{array}{c} \Delta \varphi =\delta \left(\varphi \right)\left(\mu \cdot \mathrm{div}\left(\frac{\nabla \varphi }{\left|\nabla \varphi \right|}\right)-\lambda_{1}{\left(\mu_{0}\left(x,\;\;y,\;\;z\right)-c_{1}\right)}^{2}-\lambda_{2}{\left(\mu_{0}\left(x,\;\;y,\;\;z\right)-c_{2}\right)}^{2}-v\right), \end{array}$$

where *λ*_1_, *λ*_2_, *μ*, and *v* are all weighted coefficients. *c*_1_ and *c*_2_ represent the average grayscale values inside and outside the contour, respectively, while *μ*_0_ represents the pixel gray-level value. The function *δ*(*φ*) is a Dirac delta function, which we approximate in our implementation as follows:

(2) $$\begin{array}{c} \delta \left(\varphi \right)=\frac{1}{\pi }\frac{\varepsilon }{\varepsilon^{2}+\varphi^{2}}, \end{array}$$

where *ε* is a constant that is used to control the sharpness of the contour plane. div(∇*φ*/|∇*φ*|) in (1) is used to smooth out the entire contour plane, and can be obtained from the divergence of the various pixel-gradient (∇) directions on the contour plane. As 3D-LSM increases the association between gradients on the *Z*-axis of the contour plane, it is therefore redefined as follows:

(3) $$\begin{array}{c} \mathrm{div}\left(\frac{\nabla \varphi }{\left|\nabla \varphi \right|}\right)=\frac{\gamma }{{\left(fx^{2}+fy^{2}+fz^{2}\right)}^{1.5}}\;*\;{\left(fx^{2}+fy^{2}+fz^{2}\right)}^{0.5}, \end{array}$$

where *fx*, *fy*, and *fz* represent the gradient quantization values on the *X*-, *Y*- and *Z*-axes of the image, respectively. The term *γ* can be obtained by the following formula:

(4) $$\begin{array}{c} \gamma =fy^{2}\;*\;fxx+fz^{2}\;*\;fxx+fx^{2}\;*\;fyy+fz^{2}\;*\;fyy+fx^{2}\;*\;fzz+fy^{2}\;*\;fzz-\;2\;*fx\;*\;fy\;*\;fyz-2\;*\;fx\;*\;fy\;*\;fxy-2\;*\;fx\;*\;fz\;*\;fxz. \end{array}$$

In addition, the third part of (1)—*λ*_1_(*μ*_0_(*x*, *y*, *z*) − *c*_1_)^2^ − *λ*_2_(*μ*_0_(*x*, *y*, *z*) − *c*_2_)^2^—is the key to moving the contour gradually to the edge of the object in the updating process. Through observation, it was discovered that increasing *λ*_1_ could cause the internal broken areas of the contour surface to be connected. This usually makes it easier to identify a target with lower grayscale values. Increasing *λ*_2_ can cause the external broken areas of the contour surface to be connected, which helps identify a target with higher grayscale values (e.g., the tumor area in breast MRI). The value of *v* in the fourth term of (1) is the overall height adjustment. The higher this value is, the lower the overall *φ* value will be. Meanwhile, the volume enclosed by the entire contour surface will also be reduced, so the adjustment of *v* will affect the volume inside the contour surface.

### 2.2. Three-Dimensional Surface Rendering (3DSR)

As the results obtained from 3D image segmentation are only 3D point matrices, which are not directly observable on the device, after segmentation using various methods was completed, there is a need to employ 3DSR (three-dimensional surface rendering) techniques to convert the 3D point matrix into a 3D model for viewing by users. While the 3DSR technique is not considered a part of 3D image segmentation, it is an important technique for employing segmentation algorithms in clinical applications. In general, there are two categories of 3DSR approach. The first is the isosurface approximation method [17], which attaches certain geometric planes to the equivalent surface and then uses the 3D mapping method to image the surface. The other is the light projection method [18], in which light is projected in the equidistant sampling mode for different types of accumulation. The typical techniques of 3DSR are contour-tracing isosurface (CTIS) [19], volume rendering (VR) [20], and marching-cubes isosurface (MCIS) [17, 21]. The main disadvantage of CTIS is that there may be multiple closed contours on each cross section, as well as significant differences between contour lines in two adjacent cross sections. This makes it very difficult to trace and connect the contour lines in adjacent cross sections, which might result in a large number of connection errors when tracing complex structures. The disadvantage of VR is that the projected value needs to be recalculated frequently during the process of 3D rotation, so many calculations are required. However, MCIS treats the small cubes in the 3D space as the basic units, and to find the respective isosurface of each one. The cubes can be categorized as being either inside or outside the object, based on values on the eight vertices; there are only 15 types in total, after eliminating rotationally similar states. The corresponding isosurface can then be generated quickly within the small cubes according to a lookup table. Based on the above analysis, this paper uses the MCIS method to construct a 3D model of a breast tumor. About the calculation process of MCIS, please refer to [21] for details.

### 2.3. Three-Dimensional Shape-Weighted Level Set Method (3D-SLSM)

**Figure 2:**
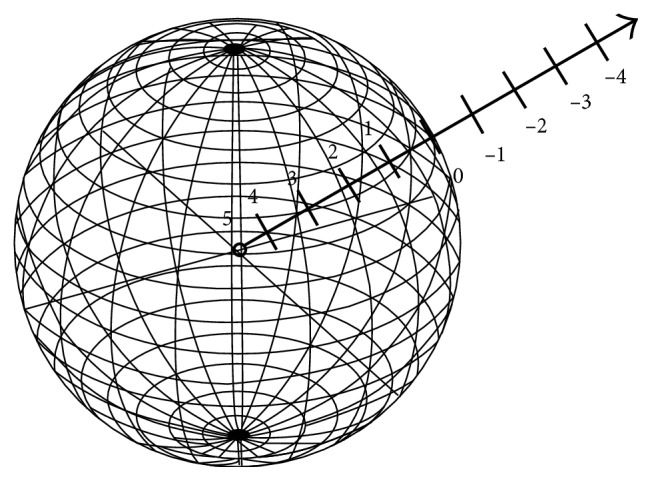
Three-dimensional shape-weighted value diagram.

In order to improve the speed and accuracy of 3D segmentation, we now combine 3D-LSM with the 3D shape characteristics to obtain the 3D shape-weighted level set method (3D-SLSM). This adds a shape-weighted value each time LSM updates the *φ* value, thus controlling the convergence of the 3D contour lines. It is important to note that the calculated *φ* value in the 3D-LSM calculation process is a 3D matrix; thus, we have to use the 3D shape characteristic to combine this *φ* value effectively with 3D-LSM. Here, a shape characteristic refers to the desired shape of the object; for different target tissues or organs in medical imaging, the expected shape can require different considerations. This paper uses a breast cancer tumor as a demonstration. In view of the usual presentation of this type of tumor as approximately spherical or ellipsoidal, the shape characteristic in the experiment is computed as a sphere. Accordingly, in each 3D-SLSM iterative operation, we must first initialize a sphere that has the same inner volume as the target. The center coordinates of the sphere locate the center of gravity for all pixels with *φ* > 0, and the volume of the sphere is equal to the sum of all such pixels. The radius of the sphere can be calculated through the sphere volume equation:

(5) $$\begin{array}{c} V=\frac{4}{3}\pi r^{3}, \end{array}$$

where *V* represents the number of pixels with *φ* > 0, *π* is the usual mathematical constant, and *r* is the radius we wish to calculate. After calculation of (5), we can obtain the radius of the sphere, and in this way, the initial characteristic shape (spherical) matrix can be established via the following equation:

(6) $$\begin{array}{c} \varphi_{s}\left(x,\;\;y,\;\;z\right)=-\sqrt{{\left(x-c_{x}\right)}^{2}+{\left(y-c_{y}\right)}^{2}+{\left(z-c_{z}\right)}^{2}}+r, \end{array}$$

where *c*_*x*_, *c*_*y*_, and  *c*_*z*_are the coordinates of the center of gravity of all pixels. After establishing the shape characteristic matrix, this is evolved further into a 3D shape-weighted value that participates in each 3D-SLSM iterative operation. Initially, the weighted value on the characteristic shape surface is defined to be zero. The farther away the interior points are from the shape surface, the greater the weighted value is. The farther away the external points are from the shape surface, the smaller the weighted value is. These two conditions combine to make the contour in the 3D-SLSM calculation process converge towards the expected characteristic shape. The 3D shape-weighted value diagram is shown in Figure 2.

**Figure 3:**
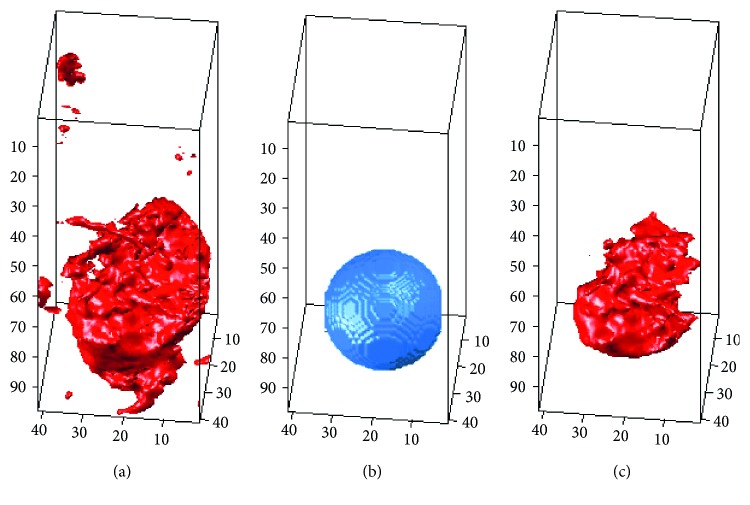
Effect of 3D shape-weighted value on a single iteration: (a) segmentation results without adding the 3D shape-weighted value; (b) calculated 3D shape-weighted value from (a); (c) segmentation results with the 3D shape-weighted value added.

**Figure 4:**
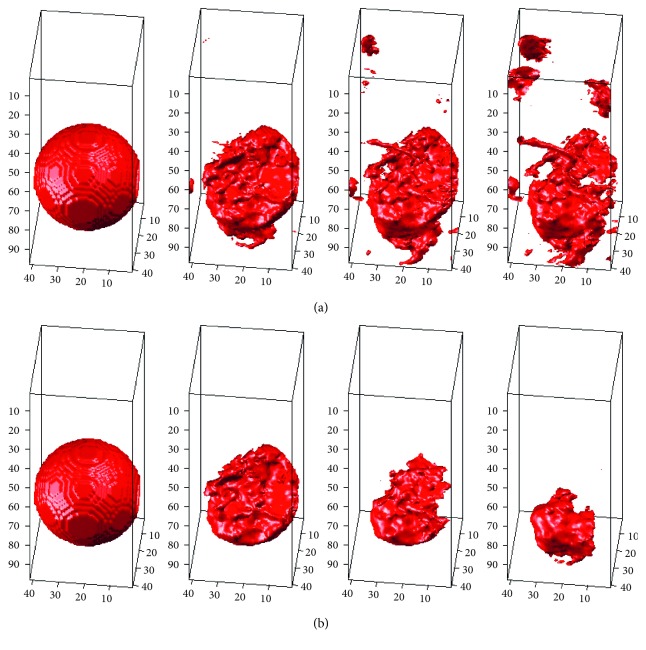
Effect of 3D shape-weighted value on the iterative calculation process: (a) partial iteration segmentation results without adding the 3D shape-weighted value; (b) partial iteration segmentation results with the 3D shape-weighted value added. Leftmost sphere is the initial contour surface in both cases.

After combining the 3D shape-weighted value, the definition of 3D-SLSM is shown in the following equation:

(7) $$\begin{array}{c} \Delta \varphi =\delta \left(\varphi \right)\left(\mu \cdot \mathrm{div}\left(\frac{\nabla \varphi }{\left|\nabla \varphi \right|}\right)-\lambda_{1}{\left(\mu_{0}\left(x,\;\;y,\;\;z\right)-c_{1}\right)}^{2}-\lambda_{2}{\left(\mu_{0}\left(x,\;\;y,\;\;z\right)-c_{2}\right)}^{2}-v+\tau \varphi_{s}\right), \end{array}$$

where *φ*_s_ is the 3D characteristic shape matrix produced by the characteristic shape, *τ* is the weighted matrix, and a combination of the two becomes the 3D shape-weighted value that controls the degree of “force” that pushes the contour towards the characteristic shape. We use an example to demonstrate the effect of the 3D shape-weighted value.  Figure 3(a) represents a 3D-LSM iterative operation. After calculating the center of gravity and the volume of Figure 3(a), we can obtain the 3D shape-weighted value diagram in Figure 3(b). Based on the 3D shaped-weighted design, the closer the points are to the center, the bigger the (positive) weight will be; the farther away the points are from the center, the smaller the (negative) weight will be. The calculated 3D-SLSM results combined with the 3D shape-weighted value are shown in Figure 3(c). Comparing Figures 3(a) and 3(c), we can see clearly in Figure 3(a) that there is a lot of surrounding noise and incomplete block fragments around the periphery of the tumor, while Figure 3(c) provides a closer look at the external appearance of the actual tumor.  Figure 4 shows the effect of the 3D shape-weighted value in the iterative calculation process. The leftmost sphere in Figure 4(a) is the initial contour surface, followed by the segmentation models of partial iterative calculation without the 3D shape-weighted value. In Figure 4(b), the leftmost sphere is the initial contour surface, followed by the segmentation models of partial iterative calculation with the 3D shape-weighted value. Comparing Figures 4(a) and 4(b), it can be observed that the 3D shape-weighted value pulls the contour towards the inside of the characteristic shape, and eliminates the impact of environmental noise and fragmented blocks around the tumor.

## 3. Experimental Data and Evaluation Methods

This paper uses breast MRI images and computer-simulated images to demonstrate three-dimensional tumor segmentation and the evaluation of its efficacy. The computer simulation is used for accurate quantitative analysis, and the actual cases aid in observing feasibility for various methods in clinical applications.

### 3.1. Establishment of Experimental Data and Evaluation Criteria for Computer-Simulated Images

**Figure 5:**
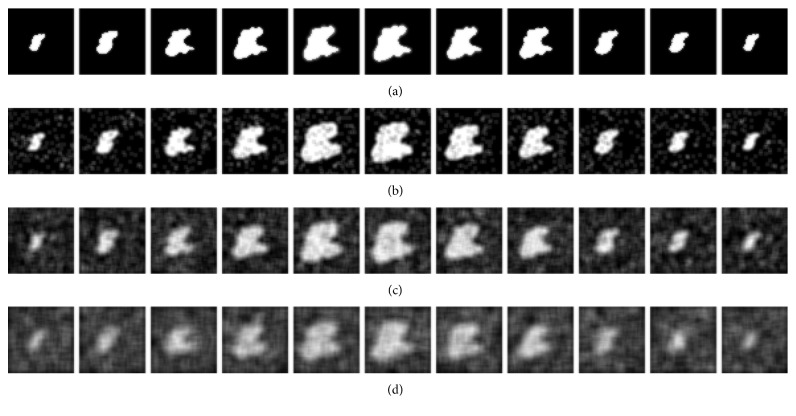
The simulated tumor images with three different levels of noise density and blurring: (a) the original image; (b) noise density at 10% with 3 × 3 mask blurring; (c) noise density at 30% with 5 × 5 mask blurring; (d) noise density at 50% with 7 × 7 mask blurring.

For the computer-simulated images to closely replicate the actual MRI images of patients with tumors, in addition to the definition of the tumor regions in the simulated images, we also added noises of varying densities (10%–50%) and blurring using masks of different sizes (3 × 3 to 7 × 7).  Figure 5 shows the original image and the 2D computer-simulated tumor images with different noise densities and levels of blurring.

### 3.2. Establishment of Experimental Data and Evaluation Criteria for Breast MRI Cases

**Figure 6:**
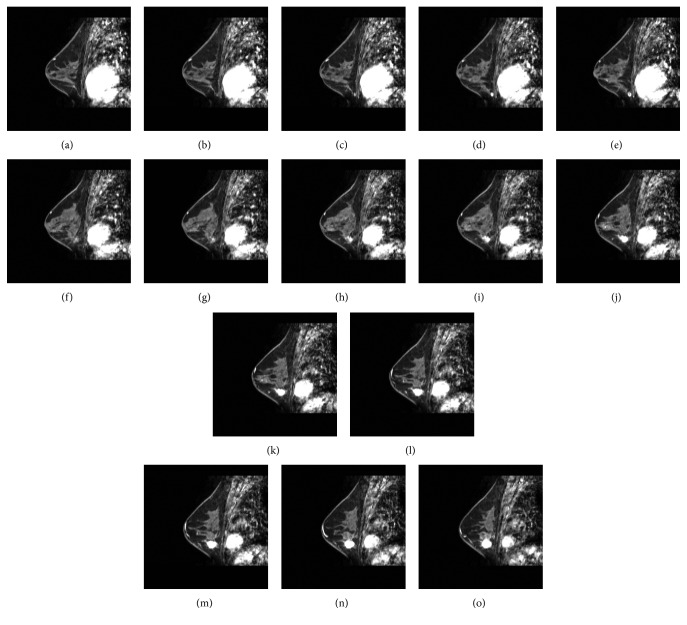
A partial image slice of an MRI case with a breast tumor.

This paper uses breast MRI as a case study for the 3D tumor segmentation experiment and performance evaluation. The imaging sources are from Taiwan Tri-Service General Hospital. The collected cases all have actual tumors, and the dynamic contrast-enhanced MRI (DCE-MRI) was performed three minutes after the injection of the developer. The resolution of each image is 512 × 512, each case has 98 image slices with a slice spacing of 2 mm, and the scope of these slices contain the tumor site.  Figure 6 shows a partial image slice of an MRI case with a breast tumor.

**Figure 7:**
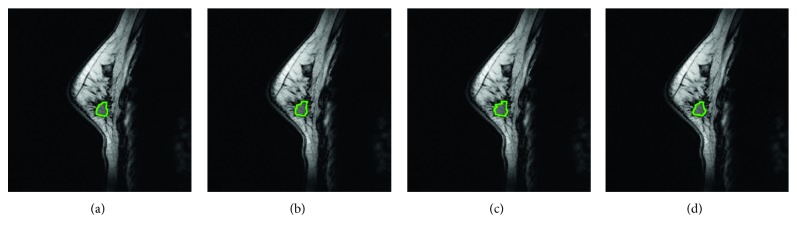
Establishment of standard tumor contour in a single slice: (a–c) tumor contour delineated by three experts; (d) contour formed by the intersection area of (a–c).

**Figure 8:**
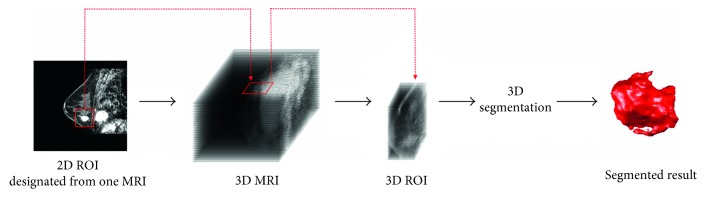
A schematic diagram for establishing a 3D ROI model based on actual MRI images.

The selection of test cases took into account the different breast sizes, tumor sizes, and breast tissue types (as shown in Table 1). In order to perform systematic quantitative evaluation, the evaluation criteria first had to be established. For slices with the tumor image in each case, three experts delineated the tumor outline. The intersection area was taken as the standard contour, and a standard 3D tumor contour was further established in combination with the standard contour in each slice. In the subsequent experiments, systematic performance evaluation and quantitative analysis was conducted for each case based on its standard 3D tumor contour.  Figure 7 shows the method of establishing a standard tumor contour in a single slice. Figures 7–7 are the tumor outlines delineated by three experts, and Figure 7(d) is the contour formed by the intersection area of the preceding three figures, that is, the standard tumor contour of that slice. The standard tumor contour in each section will be stacked in the experiment and used to construct a standard 3D tumor model. This will be used to evaluate the efficacy of various segmentation methods. In addition, the proposed system is targeted against the 3D ROI from actual cases for segmentation. The ROI region can be first delineated by the user using any 2D MRI image before the construction of a 3D ROI model. This approach facilitates the calculation of subsequent segmentation algorithms. Refer to Figure 8 for the method and process of establishing the 3D ROI.

### 3.3. Systematic Evaluation Methods

The correct classification rate (CCR), specificity (SP), and false alarm rate (FAR) are commonly used evaluation indices in a variety of medical-aided systems. The closer the values of CCR and SP to 100%, the more accurate will be the detection results of the system. However, FAR is a marker of errors that are detected from the system, and a lower percentage indicates a better performance. This work calculates the above evaluation indices based on the standard 3D tumor contour. In addition to evaluating the 3D-SLSM performance, it also compares different algorithms. Each evaluation index can be calculated by the following equations:

(8) $$\begin{array}{c} \text{correct classification rate}\left(\text{CCR}\right)=\frac{\text{TNP}+\text{TNN}}{N}, \end{array}$$

(9) $$\begin{array}{c} \text{specificity}\left(\text{SP}\right)=\frac{\text{TNN}}{\text{TNN}+\text{FPN}}, \end{array}$$

(10) $$\begin{array}{c} \text{false alarm rate}\left(\text{FAR}\right)=\frac{\text{FPN}}{N_{n}}, \end{array}$$

where *N* represents the total number of pixels in the 3D ROI and *N*_n_ represents the total number of pixels outside the standard tumor. TPN (true positive number) represents the number of pixels within the standard tumor and still remained inside the tumor after segmentation, FPN (false positive number) represents the number of pixels that were outside the standard tumor, but which were resulted as inside the tumor after segmentation, TNN (true negative number) represents the number of pixels that were initially outside the standard tumor and still remained outside the tumor after segmentation, and FNN (false negative number) represents the number of pixels that were initially inside the standard tumor, but which were resulted outside the tumor after segmentation. Generally, TPN and TNN represent the numbers of pixels segmented correctly, while FPN and FNN are the numbers of pixels wrongly segmented.

## 4. Experimental Results

### 4.1. Establishment and Comparison of 3D Tumor Segmentation Models for Computer-Simulated Images

**Figure 9:**
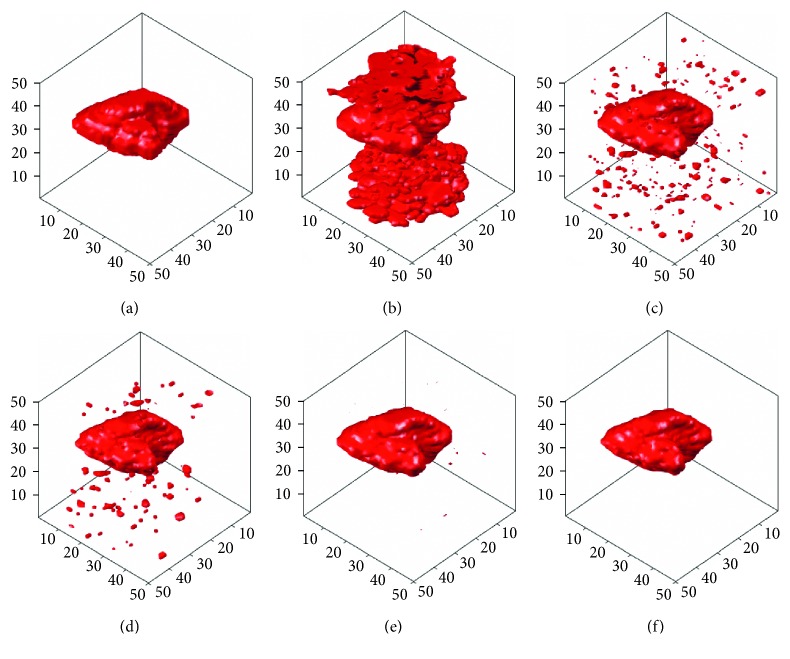
The segmentation results of computer-simulated images with 10% noise density and 3 × 3 masked blurring: (a) standard; (b) ACM; (c) LSM; (d) SLSM; (e) 3D-LSM; (f) 3D-SLSM.

**Figure 10 fig10:**
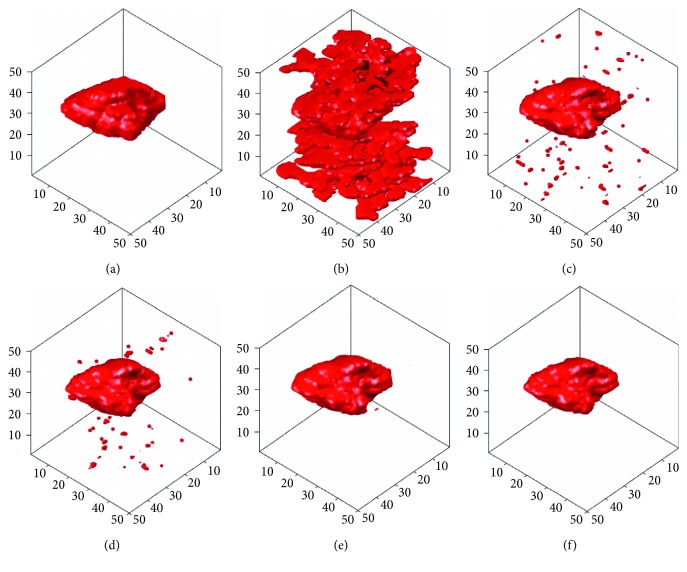
The segmentation results of computer-simulated images with 30% noise density and 5 × 5 masked blurring: (a) standard; (b) ACM; (c) LSM; (d) SLSM; (e) 3D-LSM; (f) 3D-SLSM.

**Figure 11:**
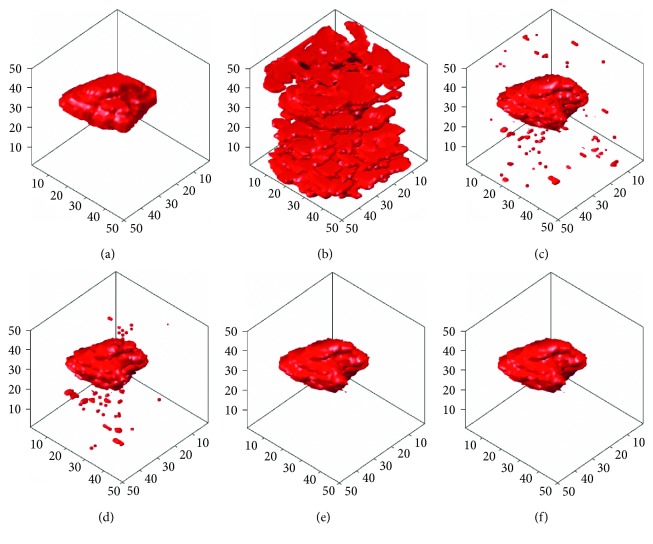
The segmentation results of computer-simulated images with 50% noise density and 7 × 7 masked blurring: (a) standard; (b) ACM; (c) LSM; (d) SLSM; (e) 3D-LSM; (f) 3D-SLSM.

In this section, the computer-simulated images that were generated in Figure 5 will be used to carry out different 3D tumor segmentation methods, and the results will be compared after the segmentation. Figures 9 [Fig fig10]–11 demonstrate the segmentation results of computer-simulated images with three different levels of blurring and noise. From the results shown in Figures 9 [Fig fig10]–11, the segmentation of 3D-SLSM from computer-simulated images, which were generated with three different levels of blurring and noise, is the nearest to standard tumors when compared with that of the existing methods (ACM, LSM, SLSM, and 3D-LSM).

### 4.2. Quantitative Evaluation and Comparison for Computer-Simulated Images

The use of computer-simulated tumor images aided in carrying out accurate quantitative analysis as the tumor region is correctly defined. Tables 2 [Table tab3]–4 show the accuracy, specificity, and the false alarm rates when a 3D tumor segmentation is carried out on computer-simulated images that have undergone three different levels of blurring (3 × 3, 5 × 5, and 7 × 7) and noise densities. From the quantitative data, we see that 3D-SLSM has the best performance in accuracy, specificity, and false alarm rates in the experimental results (Tables 2 and 3), where image blurring was carried out using different masks (3 × 3 and 5 × 5). However, when the image underwent the maximum blurring (7 × 7), 2D-SLSM and 3D-SLSM demonstrated similar performances in specificity. The overly blurred images benefitted 3D-SLSM in considering the correlation between sections to be weakened. In the same way, with maximum blurring (7 × 7) and a noise density of above 45%, 3D-LSM and 3D-SLSM delivered similar performances with regard to accuracy (as shown in Table 4). This was due to the 3D shape-weighted value not being able to carry out its function in calculation when the image is overly blurred and has too much noise.

### 4.3. Establishment and Comparison of 3D Tumor Segmentation Models for Breast MRI Cases

**Figure 12:**
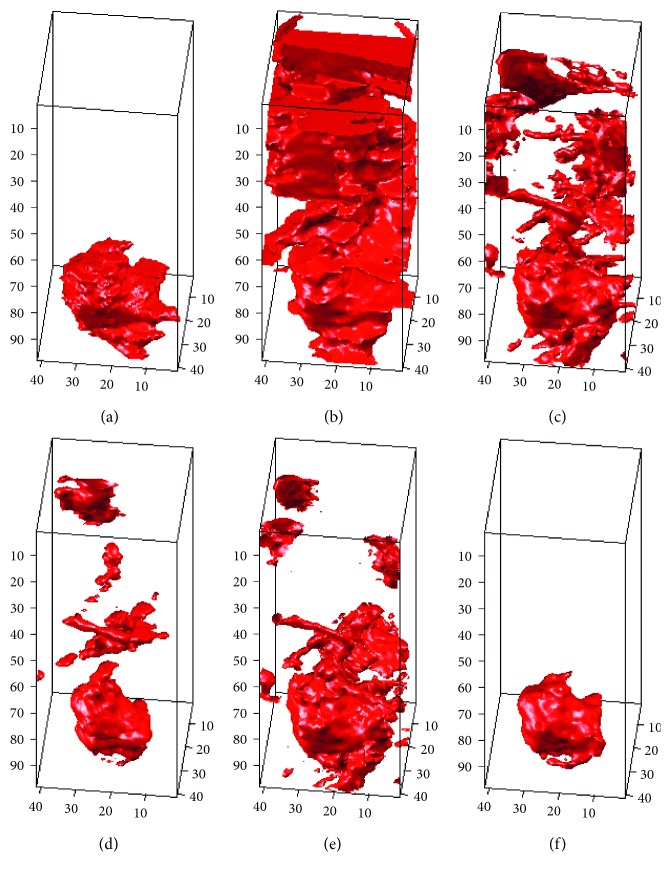
3D tumor segmentation results of Case 1 obtained with different algorithms: (a) standard; (b) ACM; (c) LSM; (d) SLSM; (e) 3D-LSM; (f) 3D-SLSM.

**Figure 13 fig13:**
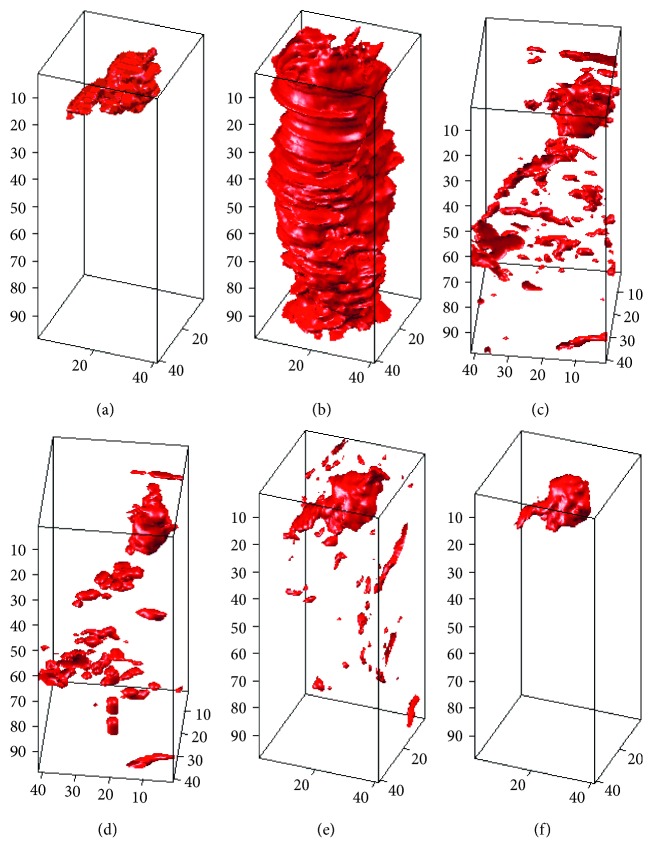
3D tumor segmentation results of Case 2 obtained with different algorithms: (a) standard; (b) ACM; (c) LSM; (d) SLSM; (e) 3D-LSM; (f) 3D-SLSM.

**Figure 14:**
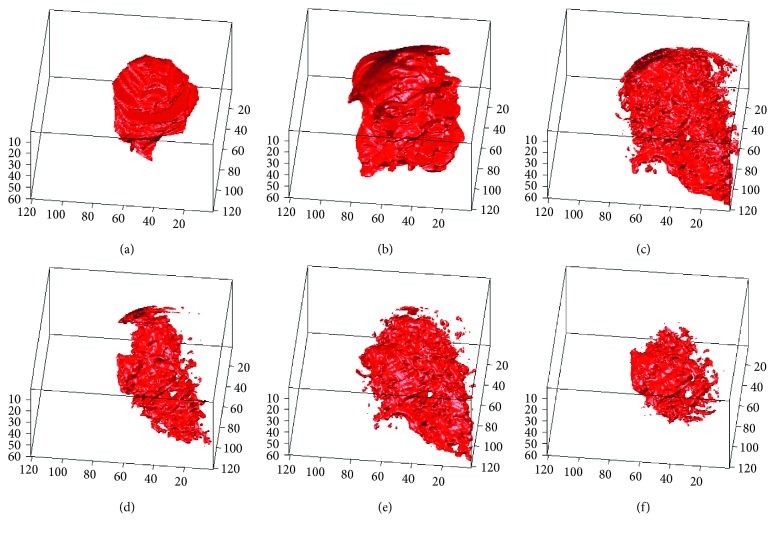
3D tumor segmentation results of Case 3 obtained with different algorithms: (a) standard; (b) ACM; (c) LSM; (d) SLSM; (e) 3D-LSM; (f) 3D-SLSM.

In this section, we use ACM, traditional LSM, shape-based LSM (SLSM), 3D-LSM, and the 3D-SLSM proposed in this paper to perform 3D tumor segmentation on the experimental cases. The resulting 3D tumor contour matrix is converted to a 3D surface (3D tumor segmentation model) via the 3DSR process of MCIS in order to observe and compare the results. The first case has a tissue type of both fat and glandular, and both the breast and tumor sizes are medium. The 3D tumor segmentation results of this case that were obtained with the various methods are shown in Figure 12. The second case has a tissue type of relatively more glandular, and both the breast and tumor sizes are relatively small. The segmentation results of this case are shown in Figure 13. The third case has a tissue type of relatively more fat, and both the breast and tumor sizes are relatively large. The segmentation results of this case are shown in Figure 14. In the experimental results of the three cases (Figures 12 [Fig fig13]–14), figure (a) is the standard 3D tumor model, the establishment process of which is described in Section 3.1; figures (b–f) are the 3D tumor segmentation models obtained by ACM, LSM, SLSM, 3D-LSM, and 3D-SLSM, respectively. The following conclusions can be drawn from these 3D tumor segmentation results. (1) When 2D segmentation techniques evolve into 3D techniques, the application of 3D segmentation gives good performance because the upper and lower slices are connected. (2) Whether it is 2D or 3D segmentation technology, combining shape characteristics can result in better performance. (3) The results in Figures 12 [Fig fig13]–14 show that the 3D tumor segmentation model generated by the 3D-SLSM proposed in this paper is the one that is most consistent with the standard 3D tumor model.

### 4.4. Quantitative Evaluation and Comparison for Breast MRI Cases

This section describes the quantitative analysis based on standard 3D tumor contours obtained from actual MRI cases (Figures 12(a), 13(a), and 14(a)). We compared the performance of 3D-SLSM with some competitive methods such as ACM, LSM, SLSM, and 3D-LSM. The construction methods employed for standard 3D tumor contours were described in Section 3.2. In addition to using the numbers of TPN, FPN, TNN, FNN, *N*_p_, *N*_n_, and *N* to show the analysis results, three evaluation markers (CCR, SP, and FAR) were calculated to facilitate the comparison of the system performance. Here, the newly added Np represents the total number of pixels inside standard tumors, and represents the actual size of the tumor.  Table 5 shows the various pixel quantities when different methods were employed to perform segmentation in different cases. Equations (8)–(10) were employed to perform the further calculation of three evaluation indicators that are commonly used in medical auxiliary systems, CCR, SP, and FAR (as shown in Table 6). Combining the observation in Tables 5 and 6, we can conclude that (1) LSM has a better performance than ACM in 3D tumor segmentation, and this confirms the appropriateness when LSM was chosen as a foundation in the proposed method. (2) The conversion of traditional 2D algorithms into 3D algorithms can increase the accuracy of 3D segmentation. (3) A combination of shape characteristics can similarly increase segmentation accuracy. (4) Converting traditional 2D-LSM into 3D algorithms and performing a simultaneous combination with shape characteristics into 3D-SLSM can obtain higher CCR and SP, as well as a lower FAR.

These quantitative results verified that 3D-SLSM can effectively eliminate background noise, so contours that are obtained from segmentation are closer to actual tumor boundaries, and demonstrate the feasibility of 3D-SLSM in clinical applications.

## 5. Conclusions

3D medical image segmentation can provide 3D information of lesions or organs, and therefore is an important auxiliary tool for medical diagnosis. However, existing 3D image segmentation techniques still have some shortcomings. Although many 2D image segmentation methods have been proven to have good results, the overlaying of results from 2D segmentation to carry out 3D image segmentation will not only result in computational burden but also poor results owing to the lack of association between adjacent sections. In order to obtain more precise 3D segmentation results and improve computational efficiency, this paper proposed an innovative 3D medical image segmentation method, which we call the 3D shape-weighted level set method (3D-SLSM). The proposed method can carry out the precise segmentation of tumors from 3D medical images. During the development of 3D-SLSM, the 2D segmentation technology was first evolved to 3D in order to facilitate the simultaneous operation of 3D MRI, with pixels associated with each other in the three coordinate axes (*X*, *Y*, and *Z*). Furthermore, since medical images often contain multiple different tissues types, the segmentation results of most of the 3D segmentation algorithms will be affected largely by errors and/or noise. Therefore, 3D-SLSM adds the 3D shape-weighted value in each iterative process according to the change in volume, which not only accelerates convergence and eliminates background noise but also brings the segmented contour closer to the actual outline of the tumor margin.

To evaluate the accuracy of 3D-SLSM, we use breast MRI cases and computer-simulated images to demonstrate 3D tumor segmentation results. The actual cases aid in observing feasibility for various methods in clinical applications and the use of computer-simulated tumor images aided in carrying out accurate quantitative analysis as the tumor region is correctly defined. For the quantitative analysis to be fairer and complete, we considered three important influencing factors (the breast volume, the tumor size, and the breast tissue type) in image segmentation for actual cases, and made serial rational arrangements for the level of noise density and blurring intensity in computer-simulated cases. As the raw results from 3D segmentation are in the form of a 3D point matrix, in order to facilitate visual observation and comparison, the 3D point matrix after segmentation was first processed by MCIS to construct a 3D tumor image model. Subsequently, the standard tumor model was used for quantitative evaluation in order to validate the performance of various algorithms. Finally, the accuracy and error rates of conventional ACM, 2D, and 3D-LSM, as well as other methods were compared. The experimental results demonstrate that the 3D-SLSM developed in this study is not only more accurate than existing methods and has less noise after segmentation, but it also has the highest accuracy and lowest false alarm rate when compared with the standard tumor model. However, it is worth noting that 3D-SLSM benefits as the overall 3D calculations decrease when the level of blurring is high, resulting in its accuracy being comparable to 2D-SLSM. When the level of blurring and noise density is simultaneously high, the 3D shape-weighted value cannot carry out its function, causing a decrease in specificity. The results of this paper may be used in the future to aid clinical diagnosis, tracking of lesions, surgical guidance, 3D shape-feature extraction, and other research.

## Figures and Tables

**Table 1:** Characteristic list of test cases.

Case number	Breast size	Tumor size	Breast tissue types
Case 1	Medium	Medium	Fatty glandular
Case 2	Small	Small	Fatty
Case 3	Large	Large	Dense glandular

**Table 2:** Accuracy, specificity, and false alarm rate (%) of the different algorithms and noise densities in 3 × 3 mask blurred computer-simulated images.

Noise density		Methods				
		ACM	LSM	SLSM	3D-LSM	3D-SLSM
5%	CCR	90.43	99.24	99.34	99.7	**99.76**
	SP	89.93	99.2	99.76	99.69	**99.96**
	FAR	10.13	0.8	0.7	0.31	**0.26**

10%	CCR	90.07	99.13	99.19	99.6	**99.67**
	SP	89.59	99.09	99.65	99.51	**99.95**
	FAR	10.52	0.92	0.85	0.42	**0.35**

15%	CCR	91.99	98.35	99.12	99.45	**99.61**
	SP	91.63	98.27	99.63	99.44	**99.94**
	FAR	8.48	1.75	0.93	0.58	**0.42**

20%	CCR	91.02	98.52	98.93	99.07	**99.52**
	SP	90.64	98.48	99.54	99.06	**99.93**
	FAR	9.51	1.56	1.13	0.98	**0.51**

25%	CCR	90.3	98.02	98.74	98.6	**99.41**
	SP	89.89	97.95	99.41	98.56	**99.92**
	FAR	10.27	2.1	1.33	1.49	**0.62**

30%	CCR	90.95	96.83	98.61	97.82	**99.32**
	SP	90.63	96.7	99.37	97.74	**99.93**
	FAR	9.58	3.35	1.47	2.31	**0.72**

35%	CCR	90.02	95.22	98.34	98.48	**99.16**
	SP	89.65	95.02	99.28	98.54	**99.91**
	FAR	10.57	5.06	1.76	1.61	**0.89**

40%	CCR	90.52	94.18	98	97.75	**98.96**
	SP	90.22	94.05	99.22	98.01	**99.91**
	FAR	10.04	6.17	2.1	2.38	**1.11**

45%	CCR	89.69	95.9	97.73	96.59	**98.7**
	SP	89.32	96.05	99.16	96.84	**99.86**
	FAR	10.92	4.35	2.4	3.61	**1.38**

50%	CCR	89.39	95.28	97.55	95.08	**98.42**
	SP	89.09	95.52	99.11	95.32	**99.85**
	FAR	11.23	5	2.6	5.22	**1.67**

**Table 3 tab3:** Accuracy, specificity, and false alarm rate (%) of the different algorithms and noise densities in 5 × 5 mask blurred computer-simulated images.

Noise density		Methods				
		ACM	LSM	SLSM	3D-LSM	3D-SLSM
5%	CCR	87.65	98.88	98.94	99.18	**99.43**
	SP	87.01	98.81	98.88	99.13	**99.9**
	FAR	13.08	1.19	1.13	0.87	**0.6**

10%	CCR	89.85	99.01	99.39	99.36	**99.38**
	SP	89.37	98.96	99.72	99.34	**99.9**
	FAR	10.74	1.04	0.65	0.68	**0.66**

15%	CCR	90.57	99.07	99.34	99.28	**99.35**
	SP	90.13	99.02	99.7	99.25	**99.92**
	FAR	9.98	0.99	0.7	0.77	**0.68**

20%	CCR	90.37	99.11	99.25	99.29	**99.39**
	SP	89.93	99.07	99.69	99.28	**99.86**
	FAR	10.2	0.94	0.8	0.75	**0.65**

25%	CCR	89.71	99.06	99.16	99.38	**99.29**
	SP	89.26	99.05	99.7	99.42	**99.91**
	FAR	10.9	0.99	0.89	0.65	**0.75**

30%	CCR	88.68	99.05	99.14	99.26	**99.15**
	SP	88.17	99.07	99.7	99.3	**99.91**
	FAR	11.98	1.01	0.92	0.79	**0.9**

35%	CCR	88.08	99.12	99.01	99.34	**98.94**
	SP	87.62	99.22	99.73	99.56	**99.96**
	FAR	12.62	0.93	1.04	0.7	**1.12**

40%	CCR	89.18	98.96	98.89	99.25	**99.31**
	SP	88.77	99.14	99.68	99.45	**99.81**
	FAR	11.46	1.1	1.17	0.8	**0.73**

45%	CCR	89.34	98.91	98.8	99.24	**99.27**
	SP	88.94	99.14	99.72	99.63	**99.74**
	FAR	11.29	1.16	1.27	0.8	**0.77**

50%	CCR	88	98.8	98.55	99.01	**99.08**
	SP	87.55	99.21	99.68	99.46	**99.76**
	FAR	12.71	1.27	1.53	1.05	**0.97**

**Table 4:** Accuracy, specificity, and false alarm rate (%) of the different algorithms and noise densities in 7 × 7 mask blurred computer-simulated images.

Noise density		Methods				
		ACM	LSM	SLSM	3D-LSM	3D-SLSM
5%	CCR	88.22	98.5	99.16	98.87	**99.21**
	SP	87.65	98.42	**99.63**	98.82	99.25
	FAR	12.48	1.59	0.89	1.2	**0.84**

10%	CCR	88.67	98.67	99.12	98.96	**99.23**
	SP	88.14	98.62	**99.66**	98.93	99.29
	FAR	11.99	1.4	0.93	1.11	**0.81**

15%	CCR	87.42	98.74	99.09	98.99	**99.19**
	SP	86.82	98.69	**99.65**	98.97	99.27
	FAR	13.32	1.33	0.96	1.06	**0.85**

20%	CCR	87.72	98.88	99.04	99.13	**99.3**
	SP	87.15	98.85	**99.68**	99.15	99.43
	FAR	13	1.19	1.02	0.92	**0.74**

25%	CCR	87.89	98.86	98.99	99.11	**99.23**
	SP	87.35	98.87	**99.69**	99.17	99.44
	FAR	12.83	1.2	1.07	0.94	**0.81**

30%	CCR	86.1	98.91	98.96	99.15	**99.23**
	SP	85.43	98.95	**99.71**	99.23	99.46
	FAR	14.72	1.16	1.11	0.9	**0.82**

35%	CCR	86.27	98.91	98.87	99.19	**99.21**
	SP	85.63	99.03	99.72	99.36	99.54
	FAR	14.54	1.15	1.2	0.86	**0.84**

40%	CCR	86.43	99	98.74	99.2	**99.21**
	SP	85.79	99.18	**99.73**	99.44	99.64
	FAR	14.37	1.05	1.34	**0.84**	**0.84**

45%	CCR	84.41	98.94	98.63	**99.16**	99.13
	SP	83.7	99.24	**99.75**	99.57	99.73
	FAR	16.51	1.12	1.45	**0.89**	0.92

50%	CCR	84.29	98.94	98.52	99.1	**99.12**
	SP	83.55	99.37	**99.78**	99.66	99.66
	FAR	16.63	1.12	1.57	0.95	**0.93**

**Table 5:** TPN, FPN, TNN, FNN, *N*_p_, *N*_n_, and *N* numbers of the different algorithms for each case in units of pixel.

	TPN	FPN	TNN	FNN	*N*_p_	*N*_n_	*N*
*Case 1*							
ACM	6768	44035	113364	571	7339	157399	164738
LSM	6695	15600	141799	644	7339	157399	164738
SLSM	5004	3074	154325	2335	7339	157399	164738
3D-LSM	6926	11959	145440	413	7339	157399	164738
3D-SLSM	6006	518	156881	1333	7339	157399	164738

*Case 2*							
ACM	5648	64123	93276	1691	2784	161954	164738
LSM	2601	4461	157493	183	2784	161954	164738
SLSM	2122	2156	159798	662	2784	161954	164738
3D-LSM	2530	2421	159533	254	2784	161954	164738
3D-SLSM	2173	253	161701	611	2784	161954	164738

*Case 3*							
ACM	57877	102006	718123	454	58331	820129	878460
LSM	55432	60356	759773	2899	58331	820129	878460
SLSM	37814	18512	801617	20517	58331	820129	878460
3D-LSM	48647	37622	782507	9684	58331	820129	878460
3D-SLSM	35338	11217	808912	22993	58331	820129	878460

**Table 6:** Accuracy, specificity, and false alarm rate (%) of the different algorithms in each case.

	CCR	SP	FAR
*Case 1*			
ACM	72.92	72.02	27.98
LSM	90.14	90.09	9.91
SLSM	96.72	98.05	1.95
3D-LSM	92.49	92.40	7.60
3D-SLSM	**98.88**	**99.67**	**0.33**

*Case 2*			
ACM	60.05	59.26	39.59
LSM	97.18	97.25	2.75
SLSM	98.29	98.67	1.33
3D-LSM	98.38	98.51	1.49
3D-SLSM	**99.48**	**99.84**	**0.16**

*Case 3*			
ACM	88.34	87.56	12.44
LSM	92.80	92.64	7.36
SLSM	95.56	97.74	2.26
3D-LSM	94.61	95.41	4.59
3D-SLSM	**96.11**	**98.63**	**1.37**
